# Serum B-cell activating factor and lung ultrasound B-lines in connective tissue disease related interstitial lung disease

**DOI:** 10.3389/fmed.2022.1066111

**Published:** 2022-12-15

**Authors:** Yukai Wang, Xuezhen Xie, Shaoyu Zheng, Guangzhou Du, Shaoqi Chen, Weijin Zhang, Jinghua Zhuang, Jianqun Lin, Shijian Hu, Kedi Zheng, Angelina Mikish, Zhuangyong Xu, Guohong Zhang, Luna Gargani, Cosimo Bruni, Anna-Maria Hoffmann-Vold, Marco Matucci-Cerinic, Daniel E. Furst

**Affiliations:** ^1^Department of Rheumatology and Immunology, Shantou Central Hospital, Shantou, Guangdong, China; ^2^Department of Radiology, Shantou Central Hospital, Shantou, Guangdong, China; ^3^Department of Ultrasound, The First Affiliated Hospital of Shantou University Medical College, Shantou, Guangdong, China; ^4^Department of Pathology, Shantou University Medical College, Shantou, Guangdong, China; ^5^Department of Surgical, Medical and Molecular Pathology and Critical Care Medicine, University of Pisa, Pisa, Italy; ^6^Division of Rheumatology, Department of Experimental and Clinical Medicine, Careggi University Hospital, University of Florence, Florence, Italy; ^7^Department of Rheumatology, Oslo University Hospital, Oslo, Norway; ^8^Unit of Immunology, Rheumatology, Allergy and Rare Diseases (UnIRAR), IRCCS San Raffaele Hospital, Milan, Italy; ^9^Division of Rheumatology, Department of Medicine, University of California, Los Angeles, Los Angeles, CA, United States; ^10^Department of Medicine, University of Washington, Seattle, WA, United States

**Keywords:** B-cell activating factor, lung ultrasound, B-lines, KL-6, high resolution CT, connective tissue disease related interstitial lung disease

## Abstract

**Objective:**

To investigate the role of serum B-cell activating factor (BAFF) and lung ultrasound (LUS) B-lines in connective tissue disease related interstitial lung disease (CTD-ILD), and their association with different ILD patterns on high resolution computed tomography (HRCT) of chest.

**Methods:**

We measured the levels of BAFF and KL-6 by ELISA in the sera of 63 CTD-ILD patients [26 with fibrotic ILD (F-ILD), 37 with non-fibrotic ILD (NF-ILD)], 30 CTD patients without ILD, and 26 healthy controls. All patients underwent chest HRCT and LUS examination.

**Results:**

Serum BAFF levels were significantly higher in CTD patients compared to healthy subjects (617.6 ± 288.1 pg/ml vs. 269.0 ± 60.4 pg/ml, *p* < 0.01). BAFF concentrations were significantly different between ILD group and non-ILD group (698.3 ± 627.4 pg/ml vs. 448.3 ± 188.6 pg/ml, *p* < 0.01). In patients with ILD, BAFF concentrations were significantly correlated with B-lines number (*r* = 0.37, 95% CI 0.13–0.56, *p* < 0.01), KL-6 level (*r* = 0.26, 95% CI 0.01–0.48, *p* < 0.05), and Warrick score (*r* = 0.33, 95% CI 0.09–0.53, *p* < 0.01), although all correlations were only low to moderate. B-lines number correlated with Warrick score (*r* = 0.65, 95% CI 0.48–0.78, *p* < 0.01), and KL-6 levels (*r* = 0.43, 95% CI 0.21–0.61, *p* < 0.01). Patients with F-ILD had higher serum BAFF concentrations (957.5 ± 811.0 pg/ml vs. 516.1 ± 357.5 pg/ml, *p* < 0.05), KL-6 levels (750.7 ± 759.0 U/ml vs. 432.5 ± 277.5 U/ml, *p* < 0.05), B-lines numbers (174.1 ± 82 vs. 52.3 ± 57.5, *p* < 0.01), and Warrick score (19.9 ± 4.6 vs. 13.6 ± 3.4, *p* < 0.01) vs. NF-ILD patients. The best cut-off values to separate F-ILD from NF-ILD using ROC curves were 408 pg/ml for BAFF (AUC = 0.73, *p* < 0.01), 367 U/ml for KL-6 (AUC = 0.72, *p* < 0.05), 122 for B-lines number (AUC = 0.89, *p* < 0.01), and 14 for Warrick score (AUC = 0.87, *p* < 0.01) respectively.

**Conclusion:**

Serum BAFF levels and LUS B-lines number could be useful supportive biomarkers for detecting and evaluating the severity and/or subsets of CTD-ILD. If corroborated, combining imaging, serological, and sonographic biomarkers might be beneficial and comprehensive in management of CTD-ILD.

## Introduction

Interstitial lung disease (ILD) is a major pulmonary complication in connective tissue disease (CTD), associated with poor prognosis and increased mortality (1, 2). The prevalence and the clinical behavior of CTD-ILD is highly variable in different rheumatic diseases, ranging from long-term stability to acute exacerbation and life threatening situations (3, 4). The unpredictable progression and outcomes make precise personalized medicine in this clinical scenario challenging. Therefore, to find sensitive, feasible, and repeatable biomarkers associated with CTD-ILD progression could be important (5–7). Although the pathophysiology of CTD-ILD is incompletely understood, immune dysregulation, and multiple pro-inflammatory cytokine may play an important role in this process (8). Local uncontrolled cytokine release probably drives the immune damage and, ultimately, may cause acute or chronic lung injury. In some circumstances this can lead to catastrophic outcomes (9). Bronchoalveolar and serological cytokine concentrations could reflect the alveolar and interstitial structural inflammation, damage, and healing. Among them, B-cell activating factor (BAFF) might play a crucial role in this pathogenesis (10). BAFF belongs to the tumor necrosis factor superfamily and is a key cytokine involved in B-cell differentiation, maturation, survival, and auto-antibody production. The overexpression of BAFF is associated with autoimmunity diseases onset and activity (11, 12), as well as a biomarker to assess response to therapy. Recent studies, most in idiopathic inflammatory myositis (IIM) patients, showed that plasma BAFF concentrations were significantly higher in ILD patients compared to those without ILD and were associated with the presence of anti-Jo-1 antibody (13, 14). These findings indicated that BAFF might be a promising biomarker for IIM-ILD.

Lung ultrasound (LUS) has been extensively used to detect parenchymal disease in the past two decades, including pneumothorax, pneumonia, pulmonary edema, and lung fibrosis (15, 16). B-lines, a comet-tail artefact, is the sonographic hallmark of ILD. B-lines number, morphology and distribution mirrored the severity and extent of interstitial involvement (17, 18). Previous studies showed B-lines number significantly correlated with high resolution computed tomography (HRCT) score, pulmonary function tests (PFTs) parameters, clinical features, and serum Krebs von den Lungen-6 Antigen (KL-6) levels (19, 20). Furthermore, multiple studies from different centers and races, consistently found LUS has excellent sensitivity and negative predictive value for CTD-ILD (21–23), compared to HRCT as the gold standard. In addition, the innate features of ultrasound, includes more feasible, user-friendly, radiation-free, and less expensive make it can play an important role in screening and follow-up (24).

However, to the best of our knowledge, the relationship between B-lines and serum BAFF levels in CTD-ILD has never been reported. In this pilot study, we investigated the inter-relationships among serum levels of BAFF, KL-6, LUS B-lines number, and HRCT Warrick score in patients with CTD-ILD, and their association with different ILD patterns on HRCT of chest, in order to primarily explore their role in the management of CTD-ILD.

## Materials and methods

### Patients and controls

Ninety-three consecutive CTD patients from the Shantou Central Hospital were enrolled, of which 63 patients were diagnosed with ILD (ILD group) and 30 patients did not have ILD (non-ILD group). Twenty-six age and sex matched healthy individuals without inflammatory, or autoimmunity disease, or pulmonary diseases were used as controls. Complete medical histories, physical examinations and laboratory data were conducted in all patients. Patients with a history of asthma, chronic obstructive pulmonary disease, idiopathic pulmonary fibrosis, lung cancer, occupational lung disease, radiation lung disease, heart failure, renal failure, children (<18 years old), and pregnancy were excluded from the study. The study was approved by the Shantou Central Hospital Ethics Committee (no. 2022-037). All investigations were conducted in compliance with the Declaration of Helsinki and all patients provided written informed consent.

### Chest high resolution computed tomography examination and assessment

High resolution computed tomography of the chest was performed in all patients using 128 multi-slice CT (SIEMENS SOMATOM Definition Flash CT, German). All patients were scanned in the supine position from the lung apex to the diaphragm during end-inspiration. The acquisition parameters were as follows: 1 mm collimation and 0.9–1.2 pitch, 120 kV tube voltage and 110 reference mAS. Edge-enhancing B70 kernel was obtained by using filtered back projection for clinical reading with lung window. No intravenous contrast agent was employed. The duration of the CT acquisition was 1–3 s. Matrix was 512 × 512, and the effective dose was in the range of 1–3 mSv. The presence and pattern of ILD were defined by HRCT findings assessed by a radiologist (25). The ILD group was further divided into fibrotic ILD (F-ILD) or non-fibrotic ILD (NF-ILD) according to the radiologic patterns including honeycombing, traction bronchiectasis, and/or volume loss (26). The Warrick score was used to assess HRCT ILD severity and extent by two experienced radiologists, who evaluated jointly while blinded to the clinical, serological, and sonographic information.

### Lung ultrasound examination and assessment

Commercially available ultrasound equipment with a 2.5–3.5 MHz cardiac sector transducer was used (Siemens Medical Solutions, Erlangen, Germany) in this study. Lung ultrasound was performed by two senior ultrasound physicians who were blinded to clinical, serological, and radiographic information about patients. Ultrasound images were obtained by recording the number of B-lines in a total of 50 scanning sites (27). The sum of B-lines yielded a score reflecting ILD extent (28). A B-line was defined as a discrete laser-like vertical hyperechoic reverberation artifact arising from the pleural line, extending to the bottom of the screen without fading, and moving synchronously with respiration (29).

### Measurement of serum B-cell activating factor and Krebs von den Lungen-6 concentration

The serum was stored at −80°C. Serum BAFF level was evaluated using Human BAFF/BLyS/TNFSF13B DuoSet ELISA and DuoSet Ancillary Reagent Kit 2 (R&D Systems, cat. nos. DY124-05 and DY008, respectively) according to the manufacturer’s instructions. The detection range for BAFF was 39.1–2,500 pg/ml. Serum KL-6 concentration (U/ml) was measured with a chemiluminescent enzyme immunoassay method (LUMIPULSE G2100, Japan) in the study population. The detection range for KL-6 was 50–10,000 U/ml.

### Statistical analysis

Differences for continuous parametrically distributed variables between ILD and non-ILD groups were analyzed by ANOVA, while non-parametrically distributed data were analyzed by Chi-square using the SPSS version 16 (SPSS, Chicago, Illinois, USA). Correlations among total B-lines number, serum BAFF and KL-6 level, and Warrick score were assessed with Pearson correlations using GraphPad Prism 5.0 (GraphPad Software Inc., San Diego, CA, USA). The optimal cut-off values were determined from a receiver operating characteristic curves (ROC) using MedCalc statistical software version 12.3.0. The sensitivity, specificity, and area under the ROC curve (AUROC) were used as diagnostic performance indicators. The optimal cutoff point was identified according to Youden tests. A *p*-value of < 0.05 was regarded as significant.

## Results

### Characteristics of the patient group

Ninety-three patients (65 women, 28 men) with a diagnosis of CTD (49 rheumatoid arthritis, 15 idiopathic inflammatory myopathies, 11 primary Sjogren’s syndrome, 9 interstitial pneumonia with autoimmune features, 4 overlap, 3 systemic sclerosis, 1 mixed connective tissue disease, and 1 undifferentiated connective tissue disease) were included in our study. Demographic, clinical, serological and radiologic data are described in Table 1.

**TABLE 1:** CTD Patients’ demographic and clinical features.

	ILD (*n* = 63)	Non-ILD (*n* = 30)	F-ILD (*n* = 26)	NF-ILD (*n* = 37)
Age (years)	59.7 ± 11.0	50.7 ± 12.9	59.4 ± 13.6	60.0 ± 8.6
Sex (female/male)	45/18	20/10	17/9	28/9
Duration of disease (years)	3.0 ± 4.0	4.2 ± 5.3	3.6 ± 4.7	2.6 ± 3.3
BMI (kg/m^2^)	22.4 ± 3.0	22.8 ± 3.8	22.2 ± 3.5	22.4 ± 2.6
**Smoking status**				
Former smoker, *n* (%)	6 (9.5)	2 (6.7)	3 (11.5)	3 (8.1)
Current smoker, *n* (%)	10 (15.9)	4 (13.3)	6 (23.1)	4 (10.8)
**Diagnosis**				
RA, *n* (%)	28 (44.4)	21 (70)	10 (38.5)	18 (48.6)
IIM, *n* (%)	13 (20.6)	2 (6.7)	5 (19.2)	8 (21.6)
IPAF, *n* (%)	9 (14.3)	0 (0)	6 (23.1)	3 (8.1)
pSS, *n* (%)	7 (11.1)	4 (13.3)	3 (11.5)	4 (10.8)
SSc, *n* (%)	3 (4.7)	0 (0)	2 (7.7)	1 (2.7)
Overlap, *n* (%)	2 (3.2)	2 (6.7)	0 (0)	2 (5.4)
MCTD, *n* (%)	1 (1.6)	0 (0)	0 (0)	1 (2.7)
UCTD, *n* (%)	0 (0)	1 (3.3)	0 (0)	0 (0)
Serum BAFF level (pg/ml)	698.3 ± 627.4	448.3 ± 188.6	957.5 ± 811.0	516.1 ± 357.5
Serum KL-6 level (U/ml)	563.8 ± 573.8	284.0 ± 132.0	750.7 ± 759.0	432.5 ± 277.5
B-lines total number	102.6 ± 91.2	N/A	174.1 ± 82	52.3 ± 57.5
Warrick score	16.2 ± 5.0	N/A	19.9 ± 4.6	13.6 ± 3.4

BAFF, B-cell activating factor; BMI, body mass index; CTD, connective tissue disease; F-ILD, fibrotic interstitial lung disease; IIM, idiopathic inflammatory myopathy; ILD, interstitial lung disease; IPAF, interstitial pneumonia with autoimmune features; KL-6, Krebs von den Lungen-6; MCTD, mixed connective tissue disease; N/A, not applicable; NF-ILD, non-fibrotic interstitial lung disease; pSS, primary Sjogren’s syndrome; RA, rheumatoid arthritis; SSc, systemic sclerosis; UCTD, undifferentiated connective tissue disease.

### Serum levels of B-cell activating factor in healthy controls, patients, and subgroup patients with fibrotic interstitial lung disease and non-fibrotic interstitial lung disease

**FIGURE 1:**
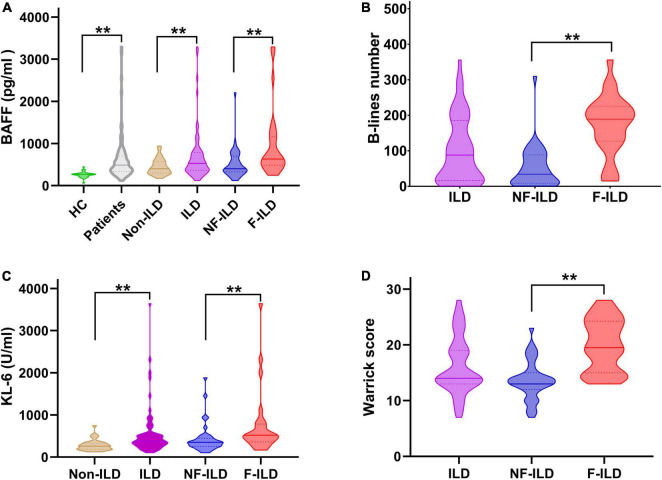
Distribution of serum **(A)** BAFF (pg/ml) and **(C)** KL-6 (U/ml) levels, **(B)** B-lines number, and **(D)** HRCT Warrick score in different groups. BAFF, B-cell activating factor; F-ILD, fibrotic interstitial lung disease; HC, healthy control; HRCT, high resolution computed tomography; ILD, interstitial lung disease; KL-6, Krebs von den Lungen-6 Antigen; NF-ILD, non-fibrotic interstitial lung disease. **P < 0.01.

Serum levels of BAFF were significantly higher in patients compared to the healthy controls (617.6 ± 288.1 pg/ml vs. 269.0 ± 60.4 pg/ml, *p* < 0.01). BAFF concentrations were significantly different when comparing the ILD to non-ILD groups (698.3 ± 627.4 pg/ml vs. 448.3 ± 188.6 pg/ml, *p* < 0.01). When patients were sub-grouped according to HRCT ILD patterns, the F-ILD had significantly higher BAFF levels compared to NF-ILD (957.5 ± 811.0 pg/ml vs. 516.1 ± 357.5 pg/ml, *p* < 0.01) (Figure 1).

### Serum levels of Krebs von den Lungen-6 in patients, and subgroup patients with fibrotic interstitial lung disease and non-fibrotic interstitial lung disease

Serum levels of KL-6 were significantly higher in patients with ILD compared to patients without ILD (563.8 ± 573.8 U/ml vs. 284.0 ± 132.0 U/ml, *p* < 0.01). In subgroup analysis, the value of KL-6 was significantly higher in patients with F-ILD vs. NF-ILD (750.7 ± 759.0 U/ml vs. 432.5 ± 277.5 U/ml, *p* < 0.01) (Figure 1).

### B-lines number and Warrick score in interstitial lung disease patients, and subgroup patients with fibrotic interstitial lung disease and non-fibrotic interstitial lung disease

In the CTD-ILD group, mean B-lines number and Warrick score were 102.6 ± 91.2 and 16.2 ± 5.0, respectively. B-lines number was significantly higher in F-ILD group compared to NF-ILD group (174.1 ± 82 vs. 52.3 ± 57.5, *p* < 0.01). F-ILD patients had more higher Warrick score compared to NF-ILD group (19.9 ± 4.6 vs. 13.6 ± 3.4, *p* < 0.01) (Figure 1).

### Correlation among serum levels of B-cell activating factor and Krebs von den Lungen-6, B-lines number and Warrick score in interstitial lung disease patients

**FIGURE 2:**
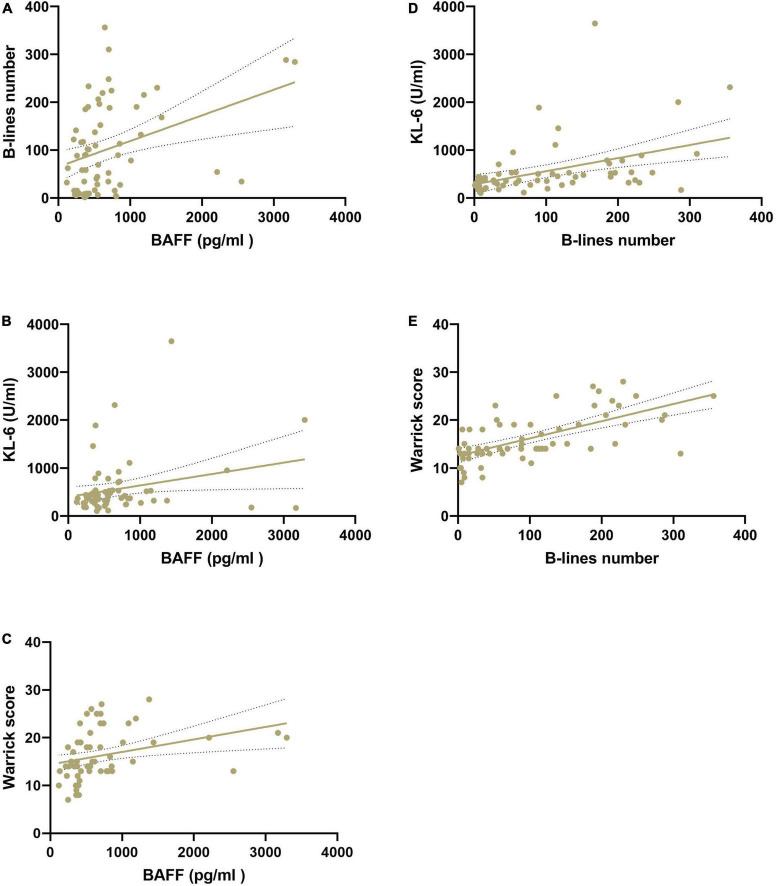
Correlation among serum BAFF (pg/ml) and KL-6 (U/ml) levels, B-lines number, and HRCT Warrick score in patients with CTD-ILD. **(A)** Correlation of serum BAFF levels and B-lines number. **(B)** Correlation of serum BAFF levels and KL-6 levels. **(C)** Correlation of serum BAFF levels and Warrick score. **(D)** Correlation of serum KL-6 levels and B-lines number. **(E)** Correlation of B-lines number and Warrick score. BAFF, B-cell activating factor; HRCT, high resolution computed tomography; KL-6, Krebs von den Lungen-6 Antigen.

In CTD patients with ILD, BAFF concentrations were statistically significantly correlated with B-lines number (*r* = 0.37, 95% CI 0.13–0.56, *p* < 0.01), KL-6 level (*r* = 0.26, 95% CI 0.01–0.48, *p* < 0.05), and Warrick score (*r* = 0.33, 95% CI 0.09–0.53, *p* < 0.01), although the correlations were low. A statistically significant positive correlation between B-lines number and the Warrick score (*r* = 0.65, 95% CI 0.48–0.78, *p* < 0.01), and KL-6 levels (*r* = 0.43, 95% CI 0.21–0.61, *p* < 0.01) was confirmed (Figure 2).

### Receiver operating characteristic analysis comparing serum B-cell activating factor and Krebs von den Lungen-6 levels, B-lines number, and Warrick score between patients with fibrotic interstitial lung disease and non-fibrotic interstitial lung disease

**FIGURE 3:**
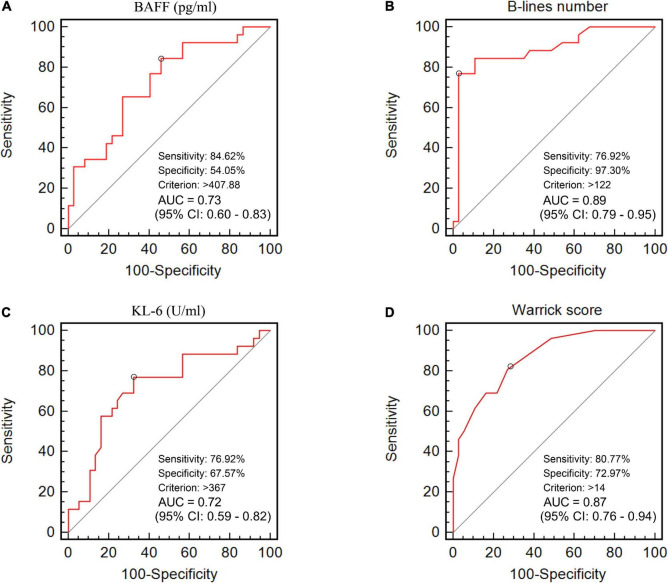
The area under the receiver operating characteristic curves of **(A)** BAFF level (pg/ml), **(B)** B-lines number, **(C)** KL-6 level (U/ml), and **(D)** HRCT Warrick score for the discrimination between F-ILD and NF-ILD.

The ROC curve analysis allowed us to use this cohort as a development cohort to define the cut-off values for BAFF and KL-6 while using these patients as a validation cohort for B-lines and Warrick scores. The B-line numbers and Warrick score separating patients with F-ILD from NF-ILD patients were 122 and 14, with areas under the ROC curve (AUC) of 0.89 for B-lines number (sensitivity 76.9%, specificity 97.3%), and 0.87 for Warrick score (sensitivity 80.8%, specificity 73%). The cut-off points for BAFF and KL-6 were 408 pg/ml and 367 U/ml, respectively while the AUC were 0.73 for serum BAFF (sensitivity 84.6%, specificity 54.1%) and 0.72 for KL-6 (sensitivity 76.9%, specificity 67.6%) (Figure 3). The relevant data are shown in Table 2.

**TABLE 2:** Receiver operating characteristic analysis of BAFF, KL-6, B-lines, and Warrick score in discriminating F-ILD from NF-ILD.

	Cut-off value	AUC	Sensitivity (%)	Specificity (%)	Accuracy (%)	*P*-value
BAFF (pg/ml)	408	0.73	84.6	54.1	66.7 (53.7–78.1)	<0.001
KL-6 (U/ml)	367	0.72	76.9	67.6	71.4 (58.7–82.1)	<0.01
B-lines number	122	0.89	76.9	97.3	88.8 (78.4–95.4)	<0.001
Warrick score	14	0.87	80.8	73	76.2 (63.8–86)	<0.001

AUC, area under curve; BAFF, B-cell activating factor; F-ILD, fibrotic interstitial lung disease; KL-6, Krebs von den Lungen-6; NF-ILD, non-fibrotic interstitial lung disease.

## Discussion

B-cell activating factor, also known as B Lymphocyte Stimulator (BLyS), is an important regulator of B-cell survival and differentiation. A growing body of evidence suggests that activation of B-cells participates in the pathogenesis of respiratory diseases, such as chronic obstructive pulmonary disease, asthma, pneumonia, and idiopathic pulmonary fibrosis by secretion of pro-inflammatory cytokine and auto-antibody (30). In bleomycin-induced lung fibrosis, genetic ablation of BAFF or BAFF neutralization by a soluble receptor significantly attenuates pulmonary fibrosis and IL-1β levels (30). In the SSc animal model, BAFF contributed to the skin and lung fibrosis by increasing IL-6-producing effector B-cells and suppressing IL-10-producing regulatory B-cells (31). Previous clinical studies found that BAFF levels significantly increased in patients with IIM-ILD compared with IIM without ILD (13). Immunohistochemistry showed BAFF was strongly expressed in patients with CTD-ILD, mainly in alveolar macrophages in the air space, parenchymal lymphoid follicles, fibroblasts and alveolar walls (32). Taken together, these results indicated that BAFF could play an important role in CTD-ILD pathogenesis.

Our study found that serum levels of BAFF in patients were significantly higher than in the healthy controls. Furthermore, we found significantly increased BAFF levels in patients with ILD compared to patients without ILD. Our findings were consistent with previous data, mostly reported from IIM cohorts. In general terms, these results showed that elevated serum BAFF level is strongly associated with ILD in CTD patients. To investigate the relationship between BAFF and different CTD-ILD patterns, the ILD group was further divided into fibrotic ILD (F-ILD) and non-fibrotic ILD (NF-ILD) (26). Subgroup analysis revealed that the serum BAFF concentration was significant higher in F-ILD than NF-ILD. Zhao et al. reported that plasma BAFF levels were significantly higher in usual interstitial pneumonia associated with autoimmune diseases and inversely correlated with PFT values (33). Our ROC analysis identified the best cut-off value of serum BAFF to discriminate F-ILD and NF-ILD was 408 pg/ml, with a sensitivity of 84.6% and a specificity of 54.1% (AUC = 0.73, *p* < 0.01). These findings indicate that BAFF levels may reflect ILD fibrotic severity and be a moderately useful biomarker for distinguishing F-ILD from NF-ILD, also suggesting that BAFF might be a new potential target for therapy in patients with CTD-ILD as well.

Belimumab, an anti-BAFF monoclonal IgG1λ antibody produced by recombinant DNA technology has been approved by the FDA for the treatment of moderate SLE patients. Recently, indications for belimumab were expanded to include lupus nephritis and juvenile lupus (34–36). Although there is no FDA indication for ILD treatment, intravenous belimumab successfully treated two cases with refractory organizing pneumonia and non-specific interstitial pneumonia related to SLE (37, 38). Relevant data are still scarce, but the rationale for belimumab treatment for CTD-ILD is increasing and it should be assessed in regard to its efficacy and safety in the future.

We found some, although low correlations between serum BAFF and KL-6 level. KL-6 is identified as a sensitive and early biomarker associated with type II alveolar epithelial cell injury, permeability, and regeneration. Elevated serum or bronchoalveolar lavage fluid KL-6 concentration could reflect incipient alveolar and interstitial inflammation (39, 40). Higher KL-6 (>1,000 U/ml) levels indicated greater mortality and worse prognosis (41). One plausible explanation for our results, showing only low correlations of these two cytokines, may be that BAFF and KL-6 reflect different phases of ILD progression. The development of ILD in CTD may be initiated through alveolar epithelial cell microinjuries (KL-6 elevation) that leads to a persistent immuno-inflammatory phase with production of cytokines (BAFF elevation), chemokines and growth factors responsible for the expansion of fibroblast and myofibroblast populations. This in turn may lead to dysregulated tissue repair, parenchyma destruction, and scarring (30, 42, 43).

In the CTD-ILD group, BAFF levels were positively correlated with lung ultrasound B-lines number (*r* = 0.37, *p* < 0.01) and HRCT Warrick score (*r* = 0.33, *p* < 0.01), although the correlations were low. This may indicate that BAFF is subject to other influences or reflects other pathogenetic mechanisms. If corroborated with further research, it indicates that BAFF concentrations may play a supportive role and would be best used in combination with other measurements.

B-lines number was significantly correlated with serum KL-6 levels (*r* = 0.47, *p* < 0.01) and HRCT Warrick score (*r* = 0.67, *p* < 0.01), replicating our previous results.

Also, B-lines number was significant higher in patients with F-ILD compared to NF-ILD. The cut-off value for B-lines to segregate the F-ILD from NF-ILD was 122. The results demonstrate that increased B-lines are associated with more severe lung fibrosis assessed by the Warrick score. To the best of our knowledge, this is the first study to investigate the relationship between lung ultrasound B-lines and BAFF in patients with CTD-ILD. The application of pulmonary parenchymal ultrasound has been highlighted and extensively performed in different clinical settings in the past two decades (44, 45). Multiple B-lines were a sensitive sign of ILD, even in the subclinical and very early stages (21, 24), more B-lines were an indicator of more severe ILD as well (17). LUS’s non-invasive, inexpensive, relatively feasible nature make it a tempting target as a screening tool (24, 46) or as a tool for follow-up of specific patients (47, 48). It could also be used in conjunction with other measurements (e g., with anti-MDA-5 antibody) for prognostic functions in patients with IIM-ILD (20, 49–51) or to support other measurements such as PFT or HRCT. The principal issue in all of these cases is a full understanding of contextual and confounding factors, requiring significant further research with multiple appropriate controls in larger trials, before adoption of LUS as a fully validated tool in CTD-ILD management.

To date, the standardization and validation of LUS examination in CTD-ILD screening and follow-up have not yet been completed. Different scoring methods and probe frequency are used in clinical operation. In addition, the calculation of the number of B-lines depends on the subjective judgment of the operator. Notwithstanding, low frequency convex probe with better penetration (more suitable for B-lines detection) and 50 scanning points (more comprehensive for B-lines assessment) applied in our study, as well as two senior ultrasound physicians cooperation, would partially help overcome the aforementioned defects.

A limitation of our data is that this is a retrospective study. Further it is from a single center with a relatively small sample size and a lack of sufficient samples across CTDs to be confident of the results for any single disease. The heterogeneous disease phenotypes, treatments and activities might affect BAFF or KL-6 levels. In addition, because approximately a third of patients failed to complete pulmonary function tests, the relationship among BAFF level, B-lines number, and PFTs variables were incomplete. Finally, the pathogenetic significance and timing of the tested cytokines are incompletely understood.

## Conclusion

In conclusion, in this pilot study, we demonstrated that BAFF levels and B-lines number are associated with CTD-ILD severity and phenotype, although correlations are low to moderate, so that these biomarkers might best be used as supportive measures with other measures of CTD-ILD. Cut-off points were proposed to separate fibrotic from non-fibrotic ILD but larger trials with more controls and diverse CTDs will be necessary before these cut-off points can be fully adapted. These findings indicate that combining serological, imaging and sonographic biomarkers could play an important role in the management of CTD-ILD in the future.

## Data availability statement

The original contributions presented in this study are included in the article/supplementary material, further inquiries can be directed to the corresponding authors.

## Ethics statement

The studies involving human participants were reviewed and approved by the Shantou Central Hospital Ethics Committee (no. 2022–037). The patients/participants provided their written informed consent to participate in this study.

## Author contributions

YW and XX: conceptualization. AM, ZX, and GZ: data curation. JL: formal analysis. YW: funding acquisition, project administration, and writing—original draft. SH: investigation. SZ and GD: methodology. KZ: resources. SC: software. DF: supervision. WZ and JZ: validation. MM-C: visualization. LG, CB, and A-MH-V: writing—review and editing. All authors have read and agreed to the published version of the manuscript.
